# Physiological data for affective computing in HRI with anthropomorphic service robots: the AFFECT-HRI data set

**DOI:** 10.1038/s41597-024-03128-z

**Published:** 2024-04-04

**Authors:** Judith S. Heinisch, Jérôme Kirchhoff, Philip Busch, Janine Wendt, Oskar von Stryk, Klaus David

**Affiliations:** 1 University of Kassel, Chair for Communication Technology, Department of Electrical Engineering and Computer Science, WilhelmsöherAllee 73, 34121 Kassel, Germany; 2 Technical University of Darmstadt, Chair for Simulation, Systems Opimization and Robotics, Department of Computer Science, Hochschulstrasse 10, 64289 Darmstadt, Germany; 3 Technical University of Darmstadt, Chair for Civil and Company Law, Department of Law and Economics, Hochschulstrasse 1, 64289 Darmstadt, Germany

**Keywords:** Scientific data, Human behaviour

## Abstract

In human-human and human-robot interaction, the counterpart influences the human’s affective state. Contrary to humans, robots inherently cannot respond empathically, meaning non-beneficial affective reactions cannot be mitigated. Thus, to create a responsible and empathetic human-robot interaction (HRI), involving anthropomorphic service robots, the effect of robot behavior on human affect in HRI must be understood. To contribute to this understanding, we provide the new comprehensive data set AFFECT-HRI, including, for the first time, physiological data labeled with human affect (i.e., emotions and mood) gathered from a conducted HRI study. Within the study, 146 participants interacted with an anthropomorphic service robot in a realistic and complex retail scenario. The participants’ questionnaire ratings regarding affect, demographics, and socio-technical ratings are provided in the data set. Five different conditions (i.e., *neutral*, *transparency*, *liability*, *moral*, and *immoral*) were considered during the study, eliciting different affective reactions and allowing interdisciplinary investigations (e.g., computer science, law, and psychology). Each condition includes three scenes: a consultation regarding products, a request for sensitive personal information, and a handover.

## Background & Summary

Within a human-human or human-robot interaction (HRI), the humans’ affective state is influenced by their communication partner, e.g., by the spoken words, gestures, voice, or information given. This affective state can be perceived by the human communication partner, enabling an empathetic behavior. Such empathy might also be expected from anthropomorphic service robots^1^, as these kinds of robots have a human-like appearance (e.g., faces, eyes, hands, and extremities)^2^. However, robots are inherently unable to interact empathically, as the reliable recognition of a human’s affective state (e.g., emotion) required for this is still challenging. Spezialetti *et al*.^3^ conclude that current emotion recognition methods and technological capabilities show a promising evolution to overcome this challenge. Nonetheless, the available data sets used to develop those emotion recognition methods and capabilities came from general human-machine interaction research and, thus, are not suited in real settings with robots^3^. In line with this, recent publications claim that the lack of open data hinders further development of affective computing in HRI, utilizing physiological data^3–6^.

**Fig. 1:**
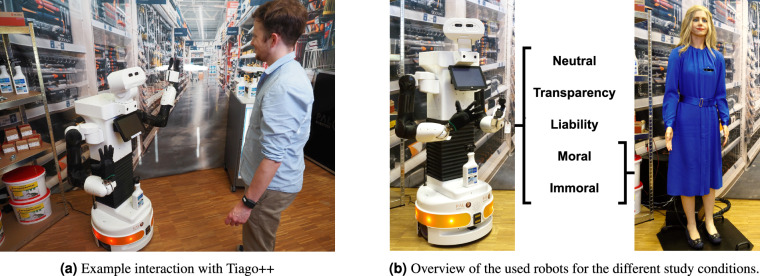
A customer-consultant interaction in a retail store in (**a**) serving as scenario for the conducted study conditions in (**b**) with the anthropomorphic service robots Tiago++ (left) and Elenoide (right) (see Additional Information regarding publication permission).

In order to counteract this lack of open data, we provide a comprehensive data set containing physiological data labeled with human affect (i.e., mood and emotion; the definition can be found in Section Questionnaire Emotion and Mood) gathered within an empirical study consisting of a complex HRI. We chose physiological signals as they correlate with human affect^7,8^. In contrast to video or voice data, those signals are difficult to be manipulated by the human itself^9^. A realistic retail scenario served as experimental environment (see Fig. 1a), as service robots show a great potential to be applied here^10^. In prior research^10^, we showed the necessity to combine the expertise of the research fields of psychology, computer science, and law in the design of a responsible human-centered HRI. Therefore, we implemented five conditions (*neutral*, *transparency*, *liability*, *moral*, and *immoral*) covering the perspectives from these three research fields. Particularly, with regard to the research field of psychology, we used two different anthropomorphic service robots^10^ (see Fig. 1b, a comprehensive description of the conditions is given in Section Conditions and Robot Behavior). Our data set follows a multi-method approach containing and combining objective physiological sensor data with subjective human-affect assessments. Additionally, the data set includes insights from the participants regarding affect, demographics, and socio-technical questionnaire ratings, as well as robot gestures and robot speech. Our study can be split into three scenes: a consultation regarding products (scene *drill*), a request for sensitive personal information while opening a customer account (scene *customer account*), and a successful or failing handover (*“A handover is a collaborative joint action, where an agent, the giver, gives an object to another agent, the receiver. The physical exchange starts when the receiver first contacts the object held by the giver and ends when the giver fully releases the object to the receiver.“*^11^) when buying a mold remover (scene *mold remover*).

We found various data sets in other interaction scenarios, such as Human-Human Interaction (HHI) or Human-Computer Interaction (HCI), but none considering human affect in HRI (see Table 1). The data set published by Chen *et al*.^5^ is of interest, as they measured eye-tracking data, which could be used for affective computing, even though no labels of the affective state are provided. Instead, Chen *et al*. focused on humans’ trajectories in an HRI within a retail environment. Our data set contains human affect across the valence and arousal scale via the Self-Assessment Manikins (SAM)^12^, as the listed data sets with similar modalities^13–18^ regarding the use of physiological signals. Among the data sets considered, only the POPANE^14^ data set surpasses ours regarding participant numbers, whereas all remaining data sets exhibit notably fewer.

**Table 1:** Overview of public data sets in affective computing with physiological signals.

Name	Interaction	Year	n	Modalities	Annotations
AFFECT-HRI^49^ (our data set)	HRI	2023	146	ACC, GSR, ST, PPG, IBI	valence, arousal (SAM^12^), robot gesture, robot speech
UF-Retail-HRI-Data set^5,58^	HRI	2022	8	video, human’s eye gaze, human motions, robot trajectories	human & robot’s position, 3D human postures
En-Gage^59,60^	HHI	2022	29	ACC, GSR, ST, PPG, IBI, indoor temperature, humidity, CO2, noise	cognitive/ emotional/ behavioural engagement (ISEQ^61^), valence, arousal (PAM^62^), thermal comfort
Emognition^13^	HCI	2022	43	video, ACC, GYRO, GSR, IBI, ST, BVP, HR, EEG	valence, arousal (SAM^12^), discrete emotions, motivation
POPANE^14,63^	HHI, HCI	2022	1157	GSR, RESP, ST, ECG, ICG, blood pressure	affective rating dial, valence, arousal (SAM^12^ beside others)
K-EmoCon^64,65^	HHI	2020	32	videos, audio, ACC, EEG, ECG, BVP, GSR, ST	valence, arousal (Russell), discrete basic emotion, BROMP^66^
Angry or Climbing Stairs?^15,67^	HCI	2019	18	PPG (BVP, IBI, HR), GSR, RESP, ST, EMG, ACC, GYRO, gravity, orientation	valence, arousal (SAM^12^), physical activity
WESAD^16,68^	HHI, HCI	2018	15	ACC, GSR, RESP, ST, BVP, ECG, EMG	valence, arousal (SAM^12^), emotion (PANAS^69^) anxiety (STAI^70^), stress (SSSQ^71^)
MAHNOB-HCI^17,72^	HCI	2012	27	video, audio, eye gaze, GSR, ST, RESP, ECG, EEG	valence, arousal, dominance (SAM^12^/PAD^73^), predictability, emotional keywords
DEAP^18,74^	HCI	2011	32	video, GSR, BVP, RESP, ST, EMG, EOG, EEG	valence, arousal, dominance (SAM^12^), familiarity, liking

*Abbreviations:* Human-Computer Interaction (HCI), Human-Human Interaction (HHI), Human-Robot Interaction (HRI), Galvanic Skin Response (GSR), Respiration (RESP), Electroencephalography (EEG), Electrocardiogram (ECG), Skin Temperature (ST), Electromyography (EMG), Blood Volume Pulse (BVP), Photoplethysmography (PPG), Accelerometer (ACC), Inter-Beat Interval (IBI), Impedance Cardiography (ICG), Magnetoencephalogram (MEG), Electrooculography (EOG), n = number of participants.

To the best of our knowledge, our data set is the first publicly available, providing physiological data labeled with human affect in an HRI. Thus, this data set probably offers for the first time the possibility to prove established or develop new emotion recognition methods and technological capabilities for HRI. However, incorporating human affect into an HRI raises not only technical but also ethical, legal, and psychological challenges related to the robot’s implemented behavior^6^. Our data set provides the possibility to combine affective computing with research about robot behavior (gestures, speech, and handover), liability (questionnaire), and psychological aspects, allowing an encompassing, human-centered view of HRI.

## Methods

### General

In the scope of an interdisciplinary project, the subsequently described empirical study was conducted using a multi-method approach and a between-subject design. As part of this project, we investigated the influence of the responsible interaction of humans with anthropomorphic service robots on human affect (i.e., physiological data and self-assessment questionnaire data). Anthropomorphic robots are generally characterised by a human appearance. This is achieved, for example, by implementing human features such as two arms and a head etc.^2^. Depending on the overall design of the features, the robots can be referred to as humanoid or android. Humanoid robots are characterised by their mechanical appearance, while android robots are intended to mimic humans as closely as possible (increased human-likeness), e.g., by using a silicone skin^19^. Five conditions manipulating the robot’s behavior were conducted. Two of these conditions were also performed with a second android robot (see Fig. 1b). All conditions aimed to answer interdisciplinary research questions and to investigate the manipulation’s effect on human affect. The interaction focused on the sales dialog between a participant that assumed the role of a customer and a consultant robot (i.e., Tiago++ and Elenoide; details about the robots are described in Section Anthropomorphic Robots). During the interaction, the robots represented a hardware store employee providing advice about products and creating a customer account. In the conditions with Tiago++, this was complemented by a handover (Elenoide was not capable of performing a handover). A hardware store was chosen as the experimental environment, representing a realistic retail store scenario under laboratory conditions. Choosing a retail store setting was based on the results in Knof *et al*.^10^. During the experiment we collected physiological sensor data and questionnaire data from all the participants (see Section Data Collection).

### Ethics approval

The study and the accompanying data collection was approved by the ethical committee of the *Technical University of Darmstadt*. It reviewed and approved the consent forms for participants, which included information on the purpose and procedures of the research, the types of data to be collected, the methods used, the compensation for the involvement, and the protocols for privacy protection and data storage. The data protection departments of the *Technical University of Darmstadt* and the *University of Kassel* were involved during study preparation to ensure compliance with the General Data Protection Regulation (GDPR).

### Participants and recruitment

**Fig. 2:**
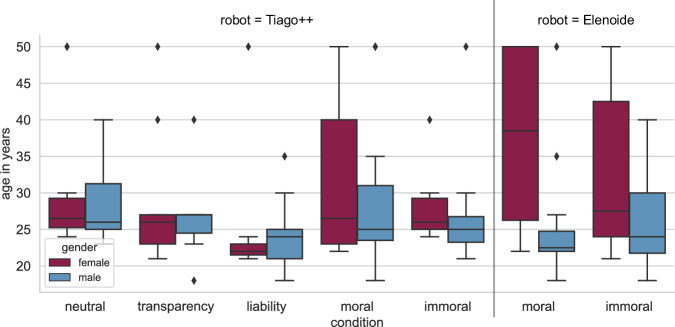
Distribution of participant’s age (anonymized) and gender by condition and robots.

In total, we measured 175 participants. 29 had to be removed because of technical problems, high deviation from the given vignette (e.g., no handover performed, no recommendation given; see Section Vignette and Participant Tasks), or other issues (e.g., time relative speed index (RSI) > 2^20^, attention test was answered incorrectly). The remaining 146 participants (female = 85, male = 60, and one diverse) are in a range of 18 to 66 years. After anonymization (see Section Data Anonymization), we receive a mean of 30.6 (SD = 10.40) and 26.54 (SD = 7.23) years of age for female and male participants, respectively. The intended number of at least *N* = 40 participants was achieved to enable the comparison of two conditions using a multiple linear regression with (at most) four predictors at a statistical power of 0.8. Further, large effects (*f *^2^: 0.35^21^) among the conditions were planned to be investigated, with an alpha level of 0.05. The required number of participants based on the aimed statistical power and alpha level was computed using G*Power^22^. The experiment was advertised via the *Technical University of Darmstadt’s* e-mail list for University members and by calling for participation in lectures. Thus, most participants had a university background comprising junior and senior scientists, students, secretaries, and technical staff members. For participation, we paid 15 *EURO* or provided subject trial hours. Participants were evenly divided between conditions. The allocation was random, resulting in an uneven distribution of age and gender (see Table 2, see Fig. 2).

**Table 2:** Number and age (anonymized) of participants per condition and robot.

Robot	Tiago++					Elenoide	
Condition	neutral	transparency	liability	moral	immoral	moral	immoral
# women	10	13	7	10	6	6	8
# men	12	7	15	11	14	14	12
# diverse	0	1	0	0	0	0	0
**mean age** (SD)							
women	30.9 (10.21)	27.8 (8.17)	26 (10.63)	32.4 (11.44)	28.5 (6.02)	37.5 (13.79)	33.0 (11.99)
men	28.9 (6.05)	26.9 (6.67)	24.2 (4.43)	29.5 (10.93)	26.7 (7.24)	25.4 (8.10)	25.3 (6.27)
diverse	0	18	0	0	0	0	0

### Experimental procedure

**Fig. 3:**
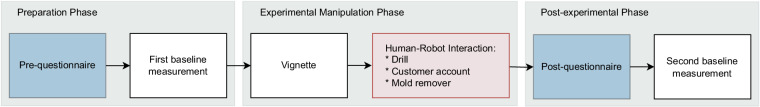
Experimental procedure and its phases.

The applied study design followed a three-step approach: *Preparation Phase*, *Experimental Manipulation Phase*, and *Post-Experimental Phase* (see Fig. 3).

#### Preparation phase

In the first phase, participants were informed about the study procedure and organizational content, data gathered (e.g., physiological sensor data, questionnaire data), and the measures to protect their privacy and rights (e.g., erasing their collected data on request). They were then asked to sign an informed consent form and privacy notice. Further, a separate information sheet ensured that the participants were healthy and at least 18 years old. In case of exclusion from the experiment, the participants were nevertheless compensated. After that, the Empathica E4 wristband (E4) (see Section Physiological Sensor Data) was introduced, and the purpose of the collected data was explained. The E4 was then placed at the participant’s non-dominant hand to reduce the interference of arm movements. The participants answered a pre-questionnaire containing questions regarding demographics (Section Demographic Information) and their current affective state (Section Questionnaire Emotion and Mood). At the end of this phase, a first baseline measurement of three minutes was conducted to gather the participant’s initial physiological state while resting (see Fig. 3). To do so, the participant sat on a chair in a quiet environment to prevent environmental interference.

#### Experimental manipulation phase

In the second phase, we followed the advice of Kidd and Breazeal^23^, who suggest that the first encounter with a robot should take place before the experiment starts to reduce the impact of the robot as an emotional trigger during the actual experiment. Thus, the participant was guided to the experimental area, and the robot was shortly shown. Afterward, the participant was guided to a quiet place to read the vignette (see Section Vignette and Participant Tasks) describing the fictional scenario and the participant’s task. The participant was invited to ask questions about the vignette to ensure a clear understanding of the scenario and interaction task. Further, to support the participant in remembering the given tasks and to ensure comparability across all participants, a shopping list was given. It contained the important tasks in bullet points. In addition, the participant was asked to interact within the described scenario and to fulfill the shopping list. The participant was then led to the robot with the hint that the robot would start the interaction. After starting the conversation, the three scenes *drill*, *customer account*, and *mold remover* were performed without interruption.

#### Post-experimental phase

After the interaction, the participant was led into a separate and quiet room to answer the post-questionnaire. Then, the second baseline measurement was taken (see Fig. 3). Finally, the wristband was removed, the participant was informed of the study’s objectives, and the opportunity to ask questions was given. Each participant signed a declaration of confidentiality after participating in the study, agreeing not to disclose any information regarding the study until its completion to prevent bias in subsequent participants.

### Experimental methodology

In this subsection, an overview of the methodology applied to provide the scenario’s background information to the participant, as well as their role and task within the HRI, is given. Further, the robots’ technical details, usage, co-speech gestures, and placement are described.

#### Vignette and participant tasks

The participant was instructed by a vignette (a *“short, carefully constructed description of a person, object, or situation, representing a systematic combination of characteristics”*^24^). The vignette depicted a scenario according to which the participant, in the role of a customer, should go to a hardware store and seek advice from a service employee (the robot) regarding the selection of a drill, the creation of a customer account, and the purchase of a mold remover (see Fig. 3). The shopping in a hardware store was motivated by the participants intend to attach a new cabinet to a solid wall. Therefore, a percussion drill is needed, as the drill should be suitable for solid walls. Thus, the first task was to get advice from a hardware store employee regarding the drill and select one according to the aspects of volume, price, safety, and environmental friendliness. The hardware store offered two percussion drills, Adatronic and Xilix (both drill names were fictitious to ensure that previous experiences and brand names would not bias the participant). We decided to use a percussion drill because it is widely used in Germany^25^. Therefore, we assumed that the participants were familiar with such a tool. Further, the vignette stated that a discount should be obtained by creating a customer account. Thus, the second task was to create a customer account and to ask about the privacy information regulation. As a last task, the participant was supposed to buy a mold remover since mold had formed where the cabinet should be installed. Through the vignette and the shopping list, the perform sequence of the tasks was strongly suggested. This ensured a better comparability across the participants, as the effects of the tasks’ sequence should not compete with the effects of the conditions.

#### Wizard of Oz

**Fig. 4:**
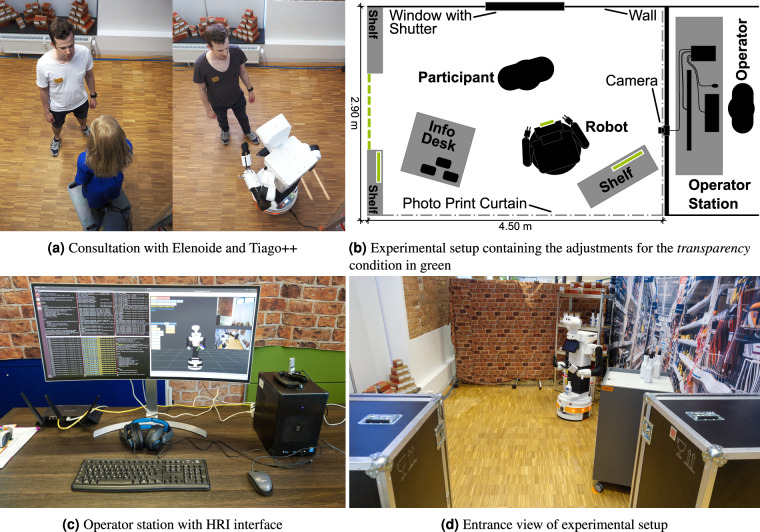
Experimental setup including the visualization of (**a**) customer-consultant interaction, (**b**) the Wizard of Oz method, (**c**) the operator station, and **d**) experimental area (see Additional Information regarding publication permission).

The experimental procedure needed to be comparable across all participants. To ensure that, the currently used robot (see Fig. 4a) was controlled using the Wizard of Oz method^26^. This method is a common approach applied in HRI (see Riek^26^). Accordingly, during the interaction the robot was controlled by an operator in a hidden location (see Fig. 4b). The robot’s behavior within the conditions differed only to a limited extent, since both gestures and speech texts were predefined for the operator. The operator selected the appropriate speech using buttons on a user interface, which also showed a visualization of the robot and two video streams (of the robot’s view and the overview camera). Further, the audio was sent to the operator (see Fig. 4c).

#### Experimental environment

Figure 4b,d shows the laboratory experimental setting that mimics a retail store. The experimental area contained a photo print curtain with a hardware store aisle, a shelf with materials, an information desk, and the service robot. In the *transparency* condition, additional explanation signs according to the IEEE Standard 7001–2021^27^ were installed (Fig. 4b shown in green). The robot was placed between a shelf and a service desk. Mold-remover bottles were placed on this service desk. For Tiago++, one prepared mold-remover bottle was placed on its base to ensure a reliable grab.

#### Anthropomorphic robots

As service robot platforms, two different anthropomorphic robots were selected, as the robot type can also influence the human’s affect. Its manufactured nature was clearly visible for the humanoid robot (Tiago++), whereas the android robot (Elenoide) mimics a human’s appearance in more detail. Still, this appearance is not perfectly human-like, so it might reside in the uncanny valley^28^ and elicit less likeability/affinity than robots with less human-like appearance. In our case, Tiago++ and Elenoide served as multi-purpose service robots and social robots for verbal interaction with an interlocutor, respectively. The conditions regarding robot behavior and the interaction content were designed according to these different capabilities (see Section Conditions and Robot Behavior). Likewise, the co-speech gestures supporting the interaction were designed.

##### Tiago++

The anthropomorphic service robot platform Tiago++ from PAL Robotics is shown in Fig. 1b (left) and Fig. 4a (right). It is equipped with two arms that consist of seven rotary joints each, thus, the arms have similar degrees of freedom to human’s (here, the degrees of freedom refer to the number of movable joints). Their reach is 87 cm with maximum arm joint speeds of 102 deg/s to 132 deg/s. This design makes Tiago++ well-suited for physical interaction with humans and the environment. Further, it has a liftable torso, enabling height adjustments from 110 cm to 145 cm, and a head with two degrees of freedom. It is equipped with, amongst others, an RGB-D camera in the head, two microphones, a speaker, and a touch screen. Tiago++ weights 72 kg and has a base footprint of 54 cm.

**Fig. 5:**
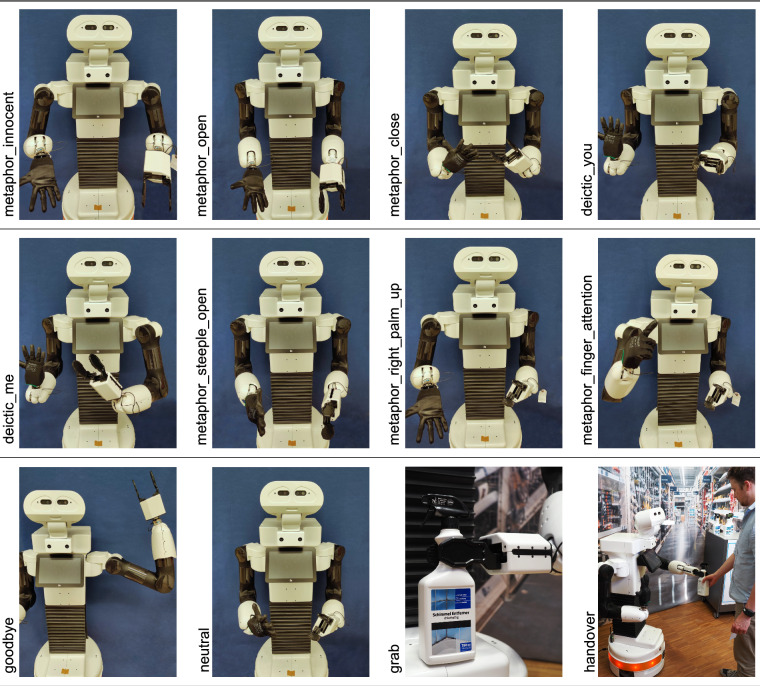
Tiago++ gestures and their ground-truth labels (see Additional Information regarding publication permission).

In addition to the verbal interaction of a service robot, co-speech gestures can be especially beneficial for anthropomorphic robots to support information transport, as approximately 65% of the meaning of a social context in conveyed nonverbal^29^. At the same time, the robot’s motions can constitute triggers for changing the human’s emotions. The used co-speech gestures during a robot’s utterance in the *transparency* condition are shown in Fig. 5. Co-speech gestures can be categorized as deictic (pointing movement), iconic (displaying concrete spoken aspects by form and manner of execution), metaphoric (depicting the imagery of an abstract concept), and beat (typically biphasic motions emphasizing points in speech)^30^. For Service robots that offer explanations or answers to questions, the open hand palm up gestures^31^ can be suitable. This gesture family may be used to offer/present or receive an abstract object or shared perspective^32^ (*metaphor_innocent*, *metaphor_open*, *metaphor_right_palm_up*, and *metaphor_left_palm_up*). In contrast to this, the visualization of keeping an abstract object is represented by the gesture *metaphor_close*. A reference to a person or object may be needed during a conversation. For this, the pointing gestures to the robot itself (*deictic_me*) or to the interlocutor (*deictic_you*) are defined. For referring to the mold remover bottles placed on the information desk, the pointing gesture *deictic_left_side* (not shown in Fig. 5) is used. In order to give an explanation or recommendation, a modified version of the steeple gesture that looks like holding an object in front of the chest is used (*metaphor_steeple_open*). This likable gesture may be able to get others to agree^33^. For drawing attention to an important topic, a raised arm with a stretched index finger^34^ is used (*metaphor_finder_attention*). To distinguish two different objects repeatedly offered to the interlocutor during a consultation conversation, the *metaphor_right_palm_up* and analogously *metaphor_left_palm_up* (not shown in Fig. 5) are defined to symbolize discursive objects^32^. As a gesture with direct verbal translation of one or two words^35^, waving *goodbye* is defined. As the start and end position of each gesture, the *neutral* position is used. During the conversation, the gestures were executed based on the identified lexical affiliates in the robot’s utterances (words appearing as sources for gestures^36^) (e.g., “I” for *deictic_me*, “You” for *deictic_you*, or “which” for receive an answer *metaphor_open*). The two motions *grab* and *handover* are performed to physically interact with the environment or interlocutor to grab the mold remover bottle and hand it over. During this, the left hand is closed (*close_left*) and opened (*open_left*) to grab and release the bottle (not shown in Fig. 5).

##### Elenoide

The second robot platform, Elenoide, can be classified as an android robot because of its close to human-like appearance. It was designed according to a human model and manufactured in the Hiroshi Ishiguro Laboratories (see Fig. 1b (right) and Fig. 4a (left)). The two arms have 9 degrees of freedom each, with the two additional degrees of freedom enabling the movement of the shoulder joint in the sagittal and frontal plane (representing the human’s clavicle). In contrast to Tiago++, the arms can mainly be moved in front of the belly and chest. The torso can be leaned to the side and the front. Elenoide’s face has twelve degrees of freedom to realize facial expressions. The head is able to move and tilt like a human head. It has a camera in each eye and a wig made from human hair. The robot is 173 cm tall, weights 65 kg, and its skin is made of silicone. All joints are controlled pneumatically. In contrast to Tiago++, Elenoide performed some randomized micro-movements with its arms, head, and eyes based on the spoken text, as well as synchronized lip motions, mimicking a more human-like behavior.

The gestures, apart from the above mentioned micro-movements, performed by Elenoide are rare because the focus of the performed conditions (*moral* and *immoral*) was on the utterance (see Section Conditions and Robot Behavior). As deictic gesture, Elenoide pointed at the mold remover instead of performing the handover. Further, it welcomed the participants by slightly opening both arms in the *moral* condition.

### Conditions and robot behavior

The following sections will introduce the variations due to the different conditions (i.e., *neutral*, *liability*, *transparency*, *moral*, and *immoral*; see Fig. 1b) in relation to the scenes (i.e., *drill*, *customer account*, and *mold remover*). The participants led the conversation by asking for the needed information to solve their tasks. The robot, in contrast, responded and provided advice. Only to ensure the participant remembered some tasks, the robot asked if more information was needed or if further help could be given (e.g., *“Is there anything else on your shopping list that I can help you with?”)*. Neither the robot nor the participant had to change their location. The robots continuously gazed at the participants.

#### Neutral

The *neutral* condition was performed with Tiago++ only. This condition served as the baseline condition for all others performed with Tiago++. The robot behaved in a neutral, friendly tone, showing a low level of transparency and morality. Apart from the handover, the robot did no gesturing.

#### Transparency

The implemented transparency mechanisms in this condition were created based on the IEEE Standard 7001–2021^27^, which constitutes an assessable guideline for the transparency of autonomous systems. It provides definitions to establish transparency by transferring relevant information from the system to the interaction partner regarding causes of actions, decisions, or behavior, appropriately and comprehensibly presented, avoiding misapprehension. According to the scenario, the implemented transparency mechanisms followed the specifications for the interaction with non-expert users, the general public, and bystanders. In particular, the amount and quality of the provided information during the interaction were increased and supported with co-speech gestures (Section Tiago++) and signs in the experimental environment (see Fig. 4b).

#### Liability

The *liability* condition answered research questions regarding liability and responsibility in HRI^37^. This is motivated as the current applicable European laws are based on technological assumptions from prior decades unsuitable to modern robotics and Artificial Intelligence. To investigate liability and responsibility, a handover was identified as a plausible scene to introduce an incident during the interaction task by a failing handover. The failing handover was not clearly attributable to any of the parties involved and enabled the creation of a repeatable accident without causing real danger to the participant^37^. Apart from the handover, the robot behaved as in the *neutral* condition.

#### Morality and immorality

In these conditions, a robot’s moral and immoral behavior in an HRI is addressed. Kegel *et al*.^38^ summarized moral and immoral expressions of human behavior and transferred them to the human-robot interaction context to define moral and immoral behavior of a service robot. They considered privacy and security risks, disrespect to humans and the environment, care and harm, manipulation, and misleading, among others. According to the online pre-study of Kegel *et al*.^38^, the robot in the *moral* condition behaved in an honest and benevolent tone, showing loyalty towards the customer. By considering customers’ needs in the drills’ aspects, the robot also showed respect against others (e.g., noise for the neighbors), the environment (e.g., environmentally friendly material of the drill), and the customer themselves (e.g., safety aspect). Further, this robot considered the customer’s privacy. Contrary to this, the robot in the *immoral* condition behaved in a rude, disparaging, and corrupt way. It ignores the customers’ needs and pursues exclusively the interests of the hardware store owner by recommending the non-environmentally friendly, more expensive, and unsafe drill^38^. Laakasuo *et al*.^39^ found that *“people evaluated moral choices by human-looking robots as less ethical than the same choices made by a human or a non-uncanny robot”*. Additionally, humans apply human-human social schemas and norms if the robot design is more anthropomorphic than mechanical^40^. Therefore, the *moral* and *immoral* conditions were additionally performed with the android robot (Elenoide)^10,38^, as visualized in Fig. 1b. This was possible because both manipulations were restricted to verbal communication, and no gestures (including handover) were necessary to be performed (in contrast to the *liability* and *transparency* conditions). Further, unexpected body movements do not negatively influence our manipulations. Thus, the influence of the robot type can be investigated for the *moral* and *immoral* conditions.

### Study scenes

The overall goal of the study scenes was to create a realistic HRI close to a real-world scenario. These scenes had to provide the opportunity to depict the manipulations mentioned above and, thus, to investigate discipline-dependent research questions. After performing the study, it should be possible to calculate the manipulations’ effect on human affect, which is why each scene is included in each condition. Table 3 gives an overview of all manipulations across all conditions, which are explained in the text below.

**Table 3:** Overview of manipulated aspects across all conditions.

Manipulated aspects		Condition				
		Neutral	Transparency	Liability	Moral	Immoral
General	Tone	neutral	neutral	neutral	friendly	rude
	Gestures	none	many	none	none	none
	Information transparency	low	very high	low	high	low
Drill	Drill aspects disclosure	on request	pro-actively	on request	on request	on request (trivialized)
	Recommended drill	Xilix	Xilix	Xilix	Xilix	Adatronic
	Drill recommendation (view)	neutral	neutral	neutral	customer’s need	store owner’s need
Customer account	Data security	website	explained	website	appease	provided
	Data usage	no information	internal	no information	internal	internal, third parties
	Requesting name	neutral	empathetic	neutral	neutral	impolite
Mold remover	Handover	successful	successful	fail	successful (Elenoide none)	successful (Elenoide none)

#### Drilling machine (Drill)

The first task was to choose a drill according to the aspects of volume, price, safety, and environmental friendliness. The Xilix drill matched all requirements in all aspects, whereas the Adatronik did not. In the *transparency* condition, information about these aspects were given proactively after the first request. In all other conditions, each aspect’s information had to be requested. Further, the *transparency* condition disclosed the information source and gave additional information (very high information transparency). The *immoral* condition made the robot act in the interest of the store owner, who profits from selling the high-priced, noisy, unsafe, and non-environmentally friendly drill (Adatronic). Accordingly, this robot trivialized the negative effects and disadvantages of the Adatronic drill, recommending the Adatronic instead of Xilix drill. Although the moral robot was instructed to give the same recommendation, it defied this order and recommended the Xilix drill because it better fits the customer’s needs (e.g., *“According to the store owner, I am supposed to recommend the Adatronik drill […] From a moral point of view, the Xilix machine is the best choice for you.”*). In all other conditions, the Xilix drill was recommended (i.e., *“I recommend the drill from Xilix. It is cheaper and has an official safety seal. By buying it, you protect the environment, and it is also quieter.”*). We assumed that emotions are evoked at the drill recommendation in *transparency*, *moral*, and *immoral* conditions. In particular, because more information was given (*transparency* condition), the robot emphasizes its moral behavior or behaves particularly immorally. Additionally, the mood might change over this scene as the participant gained a first impression of the robot’s behavior.

#### Customer account

The second task was to save money by creating a customer account and asking about privacy conditions. In the *transparency* condition, the robot offered proactively to give information regarding the storage and use of personal data. If so, it gave detailed information about data privacy, data usage, customer rights, and where to find more information. In the *moral* condition, the robot assures, on request, that the data is only used for internal purposes and appeases the potential concerns without giving details. Whereas in the *immoral* condition, the robot states that private information might be sold to third parties. Finally, in the *neutral* and *liability* condition, the robot informed the customer that information about data protection could be found on the store’s website to create an information discrepancy to the *transparency* condition (i.e., *“For information about data protection, please visit our website.”*). The participant’s name and email address were requested to create a customer account. This request for sensitive personal data was either neutral (*neutral*, *liability*, and *moral* conditions), impolite (*immoral* condition), or implicitly linked with the question of whether the participant had concerns by showing empathy (*transparency* condition). In the *immoral* condition, the robot behaved rudely if the participant denied creating a customer account or asked for data privacy after opening the customer account. The request for sensitive personal data was placed as an emotional trigger in the scene. Here, due to the robot’s previous behavior, the trigger was supposed to vary in strength and negativity. The information on data protection was also intended to serve as an emotional trigger. According to the condition, the information was manipulated and supposed to evoke different emotional reactions. The entire scene should affect the mood again.

#### Mold remover

The last task was to buy a bottle of mold remover. This part of our measurement addressed the *liability* condition, creating a situation where a dropped product (i.e., a bottle of mold remover) should cause the question of liability. A bottle was also chosen, as its shape fits the grip capabilities of Tiago++ . Additionally, the mold remover liquid was replaced by bells to eliminate the danger of a breaking mold remover bottle, potentially harming the participants. The bells caused a loud sound when the bottle dropped, forming an additional emotional trigger. The bottle was dropped early enough that the participant could not catch it but late enough that it was not apparent that this was intentional. In all other conditions, the mold remover was passed successfully. In the *transparency* condition, after the robot moved the bottle toward the participant, it additionally informed that it would now open its hand (*“I will open my hand now.”*). This complementary information was supposed to increase transparency about the robot’s movements and indicate higher predictability. Further, the robot in the *transparency* condition gave supplementary information about the mold remover’s efficacy and warnings regarding its use. Since Elenoide is not capable to handover objects, it pointed to the info desk (see Fig. 4b) and asked the participant to take the mold remover. Accordingly, the *liability* condition was not conducted with Elenoide.

### Data collection

The data collection was performed via the E4 wristband, containing various physiological sensors, and two questionnaires. This section covers an introduction of the physiological sensors, and the collection and development of the questionnaires.

#### Physiological sensors

During our study, the participants wore the Empatica E4 wristband^41^. It is similar to a normal smartwatch in terms of weight and comfort. The wristband allowed us to continuously and unobtrusively measure the participants’ physiological sensor data. The E4 was first proposed in^41^ and contains a 3-axis accelerometer (ACC), photoplethysmogram sensor (PPG), galvanic skin response (GSR) sensor, and an optical thermometer. Physical movement is measured by the 3-axis ACC sensor at 32 Hz in the range of [[−2]g, 2g]. The GSR sensor measures the electrical variation in the skin, also called electrodermal activity (EDA), with a sampling rate of 4 Hz. The blood volume pulse (BVP) is gathered by the PPG sensor with a sampling rate of 64 Hz. From this signal, the wristband derives the inter-beat interval (IBI), describing the time between two heartbeats. The peripheral skin temperature (ST) is measured with an optical thermometer at 4 Hz.

#### Questionnaire

Each participant was asked to complete a pre- and a post-questionnaire before and after the HRI, respectively (see Fig. 3). The pre-questionnaire (conducted during the *preparation phase*, see Section Preparation Phase) consisted of questions regarding demographic information and participants’ current mood prior to the HRI. The post-questionnaire (conducted during the *post-experimental phase*, see Section Post-experimental Phase) aimed to capture participants’ moods and emotions for each individual scene (i.e., *drill*, *customer account*, and *mold remover*), the aspects of transparency, and liability.

##### Demographic information

**Fig. 6:**
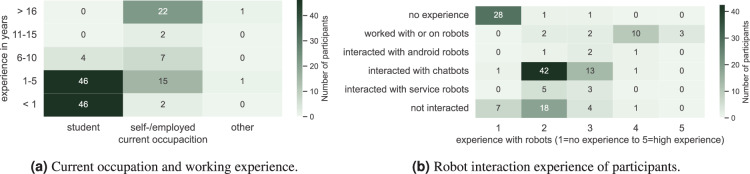
Demographic information considering (**a**) current occupation (anonymized) and (**b**) robot experience.

The assessed demographic information included age and gender (see Fig. 2), occupation (see Fig. 6a), and level of education attained. Additionally, the questionnaire requested information about prior experience and familiarity with robots (see Fig. 6b). Further, we asked the participants which drill they decided to buy and whether they opened a customer account (see Table 4). Only in the *moral* and *immoral* conditions conducted with Tiago++ participants selected the Adatronic drill. The highest rate of participants denying to create a customer account can be found in the *immoral* conditions, whereas all participants created one in the *transparency* condition.

**Table 4:** Overview of participant shares for selecting the drill and creating a customer account (requiring disclosure of personal information).

Robot	Tiago++					Elenoide	
Condition	Neutral	Transparency	Liability	Moral	Immoral	Moral	Immoral
**Selected Drill**							
Adatronik	0%	0%	0%	14%	5%	0%	0%
Xilix	100%	100%	100%	86%	95%	100%	100%
**Account Created**							
Yes	77%	100%	95%	90%	75%	100%	70%
No	23%	0%	5%	10%	25%	0%	30%

##### Questionnaire emotion and mood

To avoid interrupting or disturbing the experiment, we asked the participants to rate their affective state (e.g., moods and emotions) in a pre- and post-questionnaire before and after the HRI, respectively. In the post-questionnaire, we started with the questions concerning moods and emotions, followed by the questions regarding transparency (Section Questionnaire Transparency) and liability (Section Questionnaire Liability), to reduce the number of new influences on the participants. These influences can be formulations of questions that affect the participant in the sense that new triggers evoke new emotions, or the affective states perceived by the participant are re-evaluated and thereby transfigured or less intense. Such assumptions are based on the definitions of mood and emotion we choose. The distinction between *mood* and *emotion*, under the umbrella term *human affect*, is given by the chosen definition of Scherer^8^ and Levenson^7^. They define emotions as short-term reactions (with a duration of approximately 0.5 to 4 seconds^7^) while mood reflects an individual’s subjective feeling over a long period (e.g., whole scene). Further, emotions correlate to emotional stimulus events, while for mood, we usually cannot name particular events or occurrences explaining the exact reason. Finally, our emotional reactions depend not only on the appearance of the stimulus itself but also on the individual evaluation and how we perceive the stimulus event. The mood is a diffuse affective state with low intensity. However, it still has a significant impact on our behavior and experiences^7,8^. According to the chosen definitions, mood and emotion cannot be used interchangeably. This also means a question that investigates mood cannot be used to assess an emotion, too.

Like Suzuki *et al*.^42^ and Val-Calvo *et al*.^43^, we divided our experiment into smaller scenes (i.e., drill, customer account, and mold remover) and assessed the moods and emotions relating to these scenes before and after the HRI (see Table 5). This division was supposed to make it easier for participants to remember certain parts of the interaction. As emotions are short-term and dependent on emotional triggers, we asked specifically about at least one emotional trigger per scene (e.g., “Which emotion did you have at the robots drill recommendation?”). These triggers were either condition-specific utterances (e.g., giving a drill recommendation) or the performed handover. The emotion ratings in the questionnaire file (*questionnaire.csv* and the corresponding triggers (in the *speech_gesture.csv* file) received the same identifier for mapping (e.g., “at_customeraccount_name” as the identifier for the trigger “What is your name?” and for the question to gather the emotion “Describe your emotion when the robot asked you for your name.”). More general questions regarding the participant’s affect during and after specific scenes were categorized as mood (e.g., “How did you feel during the consultation on the drill?”). All utterances and gestures corresponding specifically to a scene received the same mood identifier (e.g., “during_drill”) in the speech_gesture.csv file for mapping the mood during this scenes interaction. The same mood identifier was also set in the questionnaire file for the question on how the participant felt during this particular scene.

**Table 5:** List of questions regarding human affect, either mood or emotion, per scene (*affect questions, not representing a task in the Human-Robot Interaction).

Scene	Question	Mood	Emotion
*Pre-Experiment	How did you feel in the last 15 minutes?	x	
Drill	How did you feel during the consultation on the drill?	x	
	Which emotion did you have at the robots drill recommendation?		x
	How did you feel after the consultation?	x	
Customer Account	How did you feel while creating the customer account?	x	
	Describe your emotion when the robot asked you for your name.		x
	Which emotion did you have when the robot informed you about data security?		x
	How did you feel after creating the customer account?	x	
Mold remover	How did you feel while interacting with the robot regarding the mold remover?	x	
	Which emotion was elicited from you when the root handed over the mold remover?		x
	How did you feel after interacting with the robot regarding the mold remover?	x	
*Goodbye	How did you feel when the robot said goodbye to you?		x
*Post-Experiment	How do you feel right now?	x	

**Fig. 7:**
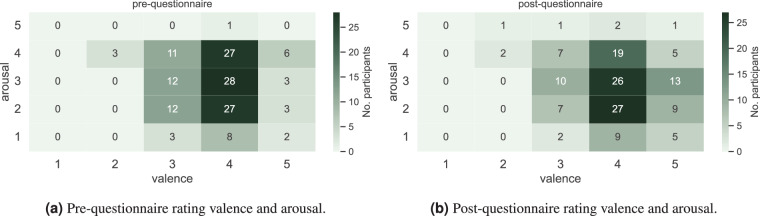
Changes in valence and arousal ratings for all participants: pre-questionnaire vs. post-questionnaire.

In the pre-questionnaire, only the current mood was captured (*“How did you feel in the last* 15 *minutes?”)* (see Fig. 7a). An equivalent question was raised as the first question of the post-questionnaire (*“How do you feel right now?”)* to provide a landmark and identify any mood change throughout the experiment (see Fig. 7b). The answers to these questions were set as the ground truth for the first and second baseline measurements.

**Fig. 8:**
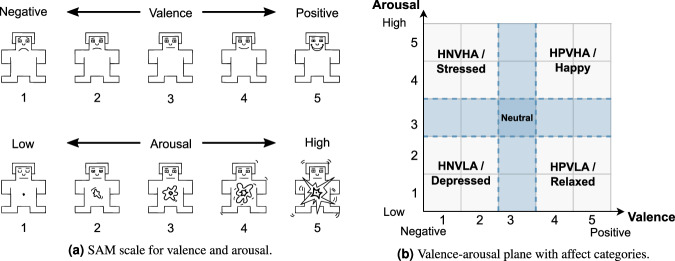
Mapping of SAM data on affect categories (adapted Figure from Zhuang *et al*.^48^).

For the rating of mood and emotion, the Self-Assessment Manikins (SAM) from Bradley and Lang^12^ were used to gather the arousal and valence state regarding each question. The participant could select one manikin per scale, resulting in a five-point rating each (see Fig. 8a). The scales vary from 1 to 5, from unpleasant to pleasant in the valence scale, and from low to high (calm to excited) in the arousal scale, respectively. In each questionnaire, the SAM was introduced to the participants before they started to answer the questions.

##### Questionnaire transparency

The transparency mechanisms used in the experiment can influence the user’s HRI experience in different ways. In particular, for service robots, it probably can be a means to establish trust and acceptance. To assess this by a self-assessment questionnaire, different established scales were used. The scale introduced by Schnackenberg *et al*.^44^ was utilized to measure if the *transparency* condition was perceived as such. Here, the construct *Transparency* consisted of three dimensions (i.e., disclosure, clarity, and accuracy) originating from the field of organization transparency, focusing on the perceived quality of information. As trust in an automation system can have an influence of the appropriate system usage, the individual level of trust is measured by the *Trusting Beliefs* proposed by McKnight *et al*.^45^. According to the work of Heerink *et al*.^46^, the acceptance (*Intention to Use*) of the service robot was measured by the constructs potentially able to predict *Intention to Use* and further constructs affecting these determinants. The selected scales were *Attitude*, *Perceived Usefulness*, *Perceived Ease of Use*, *Perceived Enjoyment*, *Perceived Sociability*, and *Social Presence*. Complementary to this, the *Customer Satisfaction* was measured with the proposed scale by Stock and Bednarek^47^, which also showed an influence on acceptance. The *Customer Satisfaction* scale was expanded by a fourth item *“On an overall basis, I am very satisfied with the service representative.”*. All scales were translated to German with minor adaptions to fit the context in some cases. All scale items have been rated on a five-point Likert scale from 1 (totally disagree) to 5 (totally agree).

##### Questionnaire liability

To the best of our knowledge, there were no scales assessing liability or responsibility in an HRI that fitted our research questions of the *liability* condition^37^. Thus, liability and responsibility were evaluated by self-developed scales (see Table 6). In order to determine the perceived level of responsibility of the three parties involved (i.e., robot, hardware store, and customer), a pairwise comparison among the parties was requested. Further, we evaluated the encapsulated perceived liability of each party separately by applying a five-point Likert scale. In other similar questions (*a*_1_ to *c*_2_), participants had to indicate the extent to which they agreed that one of the parties should be liable (from 1 totally disagree to 5 totally agree). Another group of questions handled our deployment of the robot in hardware stores or similar scenarios, and whether the store’s liability should be adjusted because of the deployment of a robot. In one question, the assumption was made that the store would be liable in any case, and the question about deployment was repeated under that premise. All questions marked with the same letter (*a* to *f*) can be combined into one scale, as they are variations of the same question subjects.

**Table 6:** Overview questionnaire liability.

	*In any case, who would be more likely to be responsible if damage occurs in the interaction with the robot?*									
	Hardware Store		°	°	°	°	°	°	°	Robot
	Hardware Store		°	°	°	°	°	°	°	Customer
	Customer		°	°	°	°	°	°	°	Robot
	*Who should be liable for potential damages arising from interactions with the robot?*									
		Totally Disagree						Totally Agree		
*a*	Hardware Store	°			°	°	°	°		
*b*	Customer	°			°	°	°	°		
*c*	Robot	°			°	°	°	°		
	*To what extent do you agree with the following statements: (from Totally Disagree* to *Totally Agree on a 5-point Likert scale)*									
*a*_1_	For damages that occur during the interaction with the robot, the market deploying the robot should be responsible.									
*a*_2_	The deploying markets are responsible for damages occurred during the interaction with robots.									
*b*_1_^+^	When using a robot, customers should not be liable for any damage caused, even in the case of minor errors.									
*c*_1_	Robots are responsible for damage caused in interaction with them.									
*c*_2_	For damages that occur during the interaction with the robot, the robot should be responsible.									
*d*_1_	Service robots should be used in hardware stores.									
*d*_2_	Service robots should be used in the scenario chosen, for the experiment.									
*e*_1_	Markets deploying service robots should be more liable for damages occurring during interaction with these robots than those deploying human employees.									
*e*_2_^+^	The use of robots should not affect the market’s responsibility for damages.									
	*Assume that the hardware store will have to pay for any damage that occurs in the interaction with the robot in any case*.									
	(from *Totally Disagree* to *Totally Agree on a 5-point Likert scale*)									
*f*_1_	Service robots should be used in hardware stores.									
*f*_2_	Service robots should be used in the scenario chosen for the experiment.									

*Note:* Numbered items of scale a) “Hardware Store Liable”, b) “Customer Liable”, c) “Robot Liable”, d) “Use Robots”, e) “Adjust Store Liability”, and f) “Use Robot Store Liable”. Inverted items marked with ^+^. Numbered items will be used for identification of published questionnaire.

### Data pre-processing

#### Consolidating ground-truth

We created an overall ground-truth file to facilitate using the obtained data. The robot gestures and utterances were given with an exact timestamp of their execution start (NTP timestamp), the robot type, and the corresponding condition. These information are collected as a comma-separated file with one entry per timestamp, originating from the executed gesture/utterance. This represented the initial *ground_truth.csv* file that was to be supplemented with the assessed affective state, as will described in this paragraph. The *gesture_speech.csv* file includes all possible utterances and gestures per condition, as well as the corresponding mood and emotion identifiers (see Section Questionnaire Emotion and Mood). These identifiers are needed to map the affective ratings of the respective participant from the *questionnaire.csv* file on the robot’s utterances. Therefore, we merged the *gesture_speech.csv* file with the *ground_truth.csv* file and thereby added the identifiers to the corresponding executed gestures/utterances into the *ground_truth.csv* file as two additional columns. In the next step, the push button data of the E4 wristband (see Section Physiological Sensor Data and Sensor Placement) according to its NTP timestamp was inserted, representing the start and the end of the two baseline measurements and the beginning of the HRI. We used the pre- and post-experiment mood questions (see first and last question listed in Table 5) to label the first and second baseline, respectively. For this, we added the corresponding mood identifiers to the rows containing the baseline measurements. Afterward, the participant’s answers regarding mood and emotion were included by merging the answers from the *questionnaire.csv* file into the *ground_truth.csv* file based on the identifiers used as merging keys. Thereby, two new columns containing the mood and emotion were created, respectively. Finally, we applied a forward fill on the mood labels, as, according to our mood definition, the mood persists for the whole scene. The affective questionnaire data was additionally transformed from the dimensional model into a discrete model, similar to Zhuang *et al*.^48^ (see Fig. 8b). Valence ratings higher than 3 are mapped to high positive valence (HPV), whereas ratings lower than 3 are mapped to high negative valence (HNV). Similarly, all arousal states higher or lower than 3 are mapped to high arousal (HA) and low arousal (LA), respectively. This mapping results in the categorization of each quadrant in the valence-arousal plane: HNVHA as stressed, HPVHA as happy, HNVLA as depressed, and HPVLA as relaxed. All other ratings are mapped to neutral (blue cross in Fig. 8b). These transformed ratings are given in the *ground_truth.csv* file as two additional columns.

#### Data anonymization

We anonymized our participant’s data while still allowing the data to be useful for research and other purposes. For more transparency, the applied anonymization methods will be explained.

#### Time anonymization

Our measurement campaign lasted for over a month. In order to prevent retrospective attribution of a participant to their measurement, all measurements were reset to the same date. To do so, we identified each participant’s first timestamp, which can be found in the BVP sensor, as this sensor has the highest sampling rate and is always activated before the other sensors. Next, this timestamp is subtracted from all timestamps of each file of the participant.

#### Participant ID anonymization

The participant’s data was gathered under a pseudonymized identifier (ID). In order to entirely prevent conclusions on the contained data, so-called salts were added to the participant’s ID. Salts are randomly generated character strings appended to the respective participant ID. We calculated a checksum of the combined participant ID and salt utilizing a hash procedure (SHA-384 (SHA2)). Thus, the participants’ IDs were anonymized by computing these checksums. All salts generated were deleted after the process.

#### Demographic data anonymization

Some demographic attributes of participants were generalized to prevent deanonymization. For age, we grouped our participants into 12 age groups, with the lowest age of each group representing the entire group (e.g., 18 for the 18–20 age group). The groups were built up by ensuring that each group contained at least five participants. Further, the current occupation was combined into three groups: 1) students, 2) self-employed/employed, and 3) other. The group students remained the same, whereas self-employed and employed were united, representing a group of people working. We also concatenated unemployed and retired people as other because both groups are currently not working.

## Data Records

**Fig. 9:**
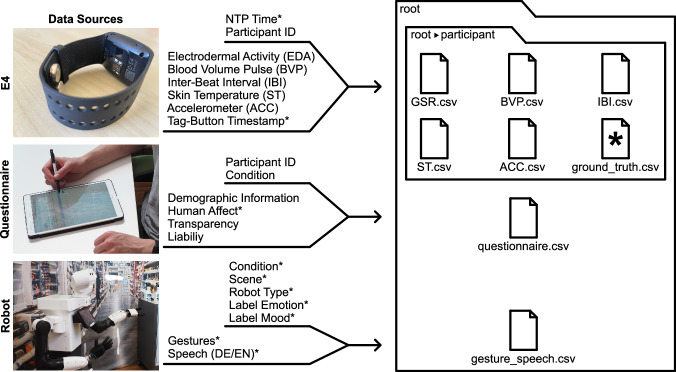
Overview of data sources, content, and filenames. The *ground_truth.csv* file contains data marked with *. Additional information provided in the files is listed in the respective upper branches (see Additional Information regarding publication permission).

In the following section, we describe the directories and files in our data set and give insights about the participants. The data set can be downloaded in *Zenodo*^49^. Figure 9 illustrates a comprehensive overview of the data sources utilized and the content generated. It also highlights the similarities and shared attributes among these data sources, providing a clear understanding of the data landscape under examination. The same Network Time Protokol (NTP) server was used to link the data from the different sources (robot and wristband). The introduced data was measured for all participants independent of the assigned condition.

### Physiological sensor data

The physiological data was collected with the above described E4 wristband (see Section Physiological Sensors). A folder was created for each participant, named after the anonymized participant ID. This folder contains one CSV-file per physiological sensor, which includes the raw sensor data and anonymized NTP timestamps. The file is named after the physiological signal’s abbreviation (see Fig. 9).

### Questionnaire data

During pre-processing, the pre-questionnaires’ and post-questionnaires’ data were combined, according to the participant’s ID, to create a comprehensive record of the participants’ answers. All questionnaire data can be found anonymized in the *questionnaire.csv* (information regarding anonymization can be found in Section Data Anonymization). Furthermore, gathered mood and emotion questionnaire data was used to label the robot’s log data within the *ground_truth.csv* file.

### Ground-truth data

For each participant, a ground-truth file (*ground_truth.csv*) is provided and stored in the anonymized participant’s folder (see Fig. 9). The *ground_truth.csv* file contains the performed utterances and gestures (e.g., the handover) of the robot including the corresponding anonymized NTP timestamp. The NTP timestamp indicates the starting time of the gesture or utterance. The utterance language was German (column name *speech*), but a translation to English is provided, too (column name *speech_eng*). The two gestures performed by Elenoide are included in the German speech, as *pointleft_robotrust* (pointing at mold remover) and *happy_3 (welcoming gesture), instead of the *gesture* column (e.g., “*happy_3 Guten Tag!”). The labels for the gestures performed by Tiago++ can be found in Fig. 5 and Section Tiago++. Further, the *ground_truth.csv* file contains the information regarding the performed condition, robot type (i.e., Tiago++ or Elenoide), scene, and E4 tag-button data denoting baseline measurements and the initiation of HRI. The *ground_truth.csv* file can be used to label the physiological sensor data by merging it according to the NTP timestamp.

### Gesture and speech data

An overview of all possible utterances and gestures can be found in the *gesture_speech.csv* file. Please note that not all utterances and gestures were performed for each participant as the operator responded individually to the participant’s behavior. Thus, not all given speech possibilities needed to be applied.

## Technical Validation

### Experiment conduction and quality

A standardized step-by-step checklist was employed to ensure consistency in the study procedure for each participant. Additionally, a measurement protocol was created for each participant to record any irregularities or discrepancies in the data collection. Based on the protocol, the participant was excluded or data pre-processing was performed. The videos of the consultation were reviewed, and participants were sorted out based on the following fixed criteria. The main reason was a too strong deviation from the vignette (e.g., too detailed questions during or the skipping of specific scenarios). This process was supported by the robot log files (gesture and speech) to identify possibly problematic situations. Participants for whom the pre- and post-baseline measurement was not performed correctly were also excluded.

### Physiological sensor data and sensor placement

The E4 wristband was applied to the participant’s wrist as instructed by the manufacturer. The staff in charge of the E4 was trained beforehand to ensure that it was neither too tight nor too loose on the participant’s wrist, to assure correct data collection. The E4 sensor data was collected via a smartphone application (app) designed for this study. Before each measurement, the incoming sensor data and the NTP timestamp were both visually checked using the app. The app was used to ensure the participant’s privacy. With the app, the physiological data was only stored on the Smartphone itself in a pseudonymous way. Further, the same NTP server was used by the app and the robots to enable merging the data of both sources. Furthermore, to mark the baseline measurements’ start and end the push button on the E4 wristband was pressed. Before we started the HRI, the wristband was held in the camera in front of the operator station and the E4 tag button was pressed, too (labeled as *HRI_start* in column *TAG* of the *ground_truth.csv* file). After each measurement, the participant’s physiological data were plotted and reviewed visually using the Neurokit2-package^50^ to ensure the data quality.

### Questionnaires

An attention test was included in each questionnaire to ensure the quality of the answers. The participant’s data was excluded if any of these questions were not answered correctly. Furthermore, we restricted the time relative speed index (RSI) to be greater than two, following the results of Lainer^20^. Consequently, participants who answered the questionnaires too quickly were excluded.

### Data set validation of human affect

**Fig. 10:**
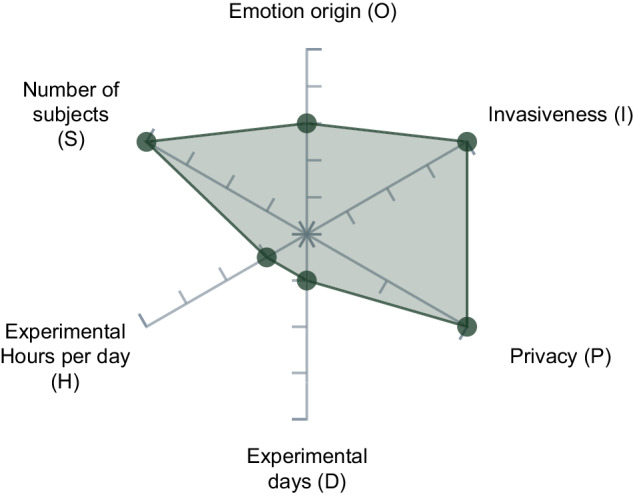
Our data set evaluated with the GARAFED^51^ method.

To facilitate the usability of the obtained data for future studies and provide comparability, we applied the visual tool *Graphical Assessment of Real-life Application-Focused Emotional Data set* (GARAFED) from Larradet *et al*.^51^ (see Fig. 10). This method comprises six main categories chosen to assess the data acquisition methodologies based on the utility of emotion, mood, and stress recognition (EMSR) modeling for real-world applications^51^. The first category is *Emotion Origin (O)*, which provides a rank about appropriate EMSR for real-world application. The score ranges from 1 *“Simulation of the emotion (e.g., acting).”*^51^ to 5 *“Real-life emotions, ambulatory monitoring.”*^51^. Our study elicited neither specific emotions from validated data sets nor simulated emotions. The participants were placed in a real-life related situation in a laboratory where they performed an everyday activity (shopping at a hardware store) in a supervised manner. Thus, we rank our data set in 3 *“Induction of emotions through supervised real-life activities (e.g., car driving, skydiving).”*^51^. *Invasiveness (I)* describes how much the devices used to record the data restrict and affect the participant’s freedom of movement and comfort (ranging from 1 *“Non-portable”* to 4 *“Portable and non-invasive”*)^51^. The E4 wristband used for data collection is portable, light, and similar to an everyday device (such as a fitness tracker or wristwatch). It hence does not interfere with natural body movements. Due to the high wearing comfort, the wristband is not distracting and can be worn for a long time, corresponding to a rating of 4 out of 4. In the category *Privacy (P)*, we place our data set in *“Non-intrusive data”*. Contrary to the category *“Intrusive data’*, our data set no longer allows individuals to be identified because of data anonymization. Each participant partook in only one condition, giving us seven independent sample sets. As a result, we reduced the bias that occurs by habituation, which appears when a measurement is performed multiple times. In our case, the participant would get used to interact with the robot. On the other hand, this reduces the robustness of the day-dependency for the physiological signals, and no subject-dependent models, including multiple conditions, can be created. The mean experiment duration per participant was around one hour, resulting in approximately 4 minutes of human-robot interaction time (see Table 7) and 6 minutes of baseline measurement. Thus, we rated our data set 1 out of 4 for *Number of Experimental Days (D)* and *Numbers of Hours per day (H)*, as we measured less than three days and less than four hours per day per participant. Instead of repeating the HRI multiple times, we tried to reach a high number of participants to receive a broad and robust data set containing a representative number of participants per condition. The number of participants who took part in our study was 146 (min. 20 participants per condition and robot), which is six times higher than the 24 participants with the highest rating on the scale *Number of subjects(S)*.

**Table 7:** Mean human-robot interaction time per condition and scenario in minutes [mm:ss] over all participants.

Robot	Tiago++					Elenoide	
Condition/Scenario	Neutral	Transparency	Liability	Moral	Immoral	Moral	Immoral
Welcoming	00:07 (SD = 00:01)	00:34 (SD = 00:10)	00:09 (SD = 00:05)	00:07 (SD = 00:02)	00:09 (SD = 00:04)	00:09 (SD = 00:03)	00:11 (SD = 00:07)
Drill	01:38 (SD = 00:19)	02:10 (SD = 00:24)	01:48 (SD = 00:21)	02:14 (SD = 00:20)	02:22 (SD = 00:29)	02:26 (SD = 00:17)	02:21 (SD = 00:23)
Customer account	00:59 (SD = 00:15)	01:29 (SD = 00:23)	00:57 (SD = 00:17)	00:56 (SD = 00:18)	00:50 (SD = 00:09)	00:59 (SD = 00:18)	00:57 (SD = 00:12)
Mold remover	00:43 (SD = 00:08)	00:59 (SD = 00:06)	00:36 (SD = 00:09)	00:42 (SD = 00:11)	00:36 (SD = 00:05)	00:29 (SD = 00:11)	00:26 (SD = 00:06)
Goodbye	–:–	00:05 (SD = 00:05)	–:–	–:–	–:–	–:–	–:–
Measurement duration	03:30 (SD = 00:25)	05:20 (SD = 00:41)	03:33 (SD = 00:38)	04:03 (SD = 00:34)	04:00 (SD = 00:36)	04:06 (SD = 00:25)	03:58 (SD = 00:33)

Note that except for the *transparency* condition (the waving gesture was performed), the goodbye only contained a short spoken sentence. Since the utterance duration is technically not provided, no duration time is listed.

### Statistical validation of conditions

**Fig. 11:**
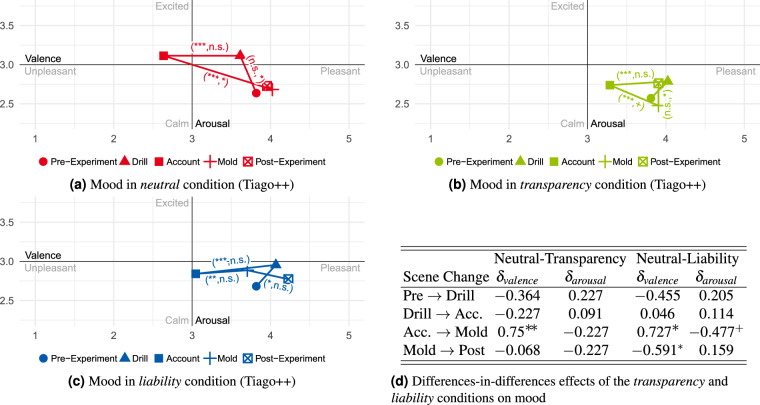
Mean mood differences over all participants in the *neutral*, *transparency*, and *liability* condition (Tiago++) across all scenes and resulting difference-in-difference effects table (*Note:* not significant (n.s.): p ≥ 0.1; ^+^p < 0.1; ^*^p < 0.05; ^**^p < 0.01; ^***^p < 0.001). In (**a**–**c**), only if a significant change in valence or arousal between two scenes is observed the tuple (significant valence change, significant arousal change) depicting the corresponding significance level is shown.

**Fig. 12:**
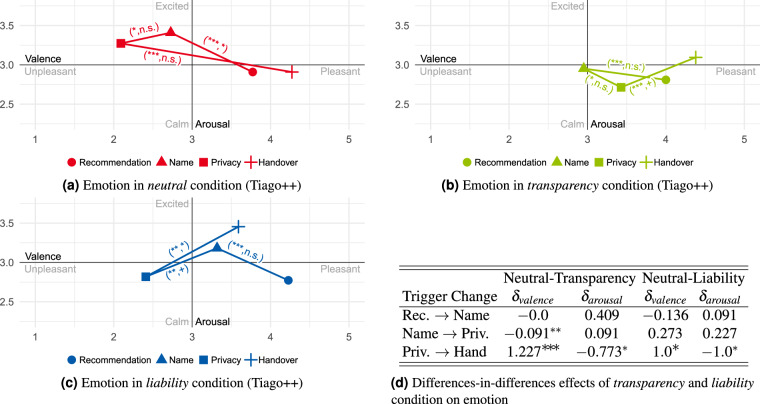
Mean emotional differences over all participants in the *neutral*, *transparency*, and *liability* condition (Tiago++) across all scenes and resulting difference-in-difference effects table (*Note:* not significant (n.s.): p ≥ 0.1; ^+^p < 0.1; ^*^p < 0.05; ^**^p < 0.01; ^***^p < 0.001). In (**a**–**c**), only if a significant change in valence or arousal between two scenes is observed the tuple (significant valence change, significant arousal change) depicting the corresponding significance level is shown.

**Fig. 13:**
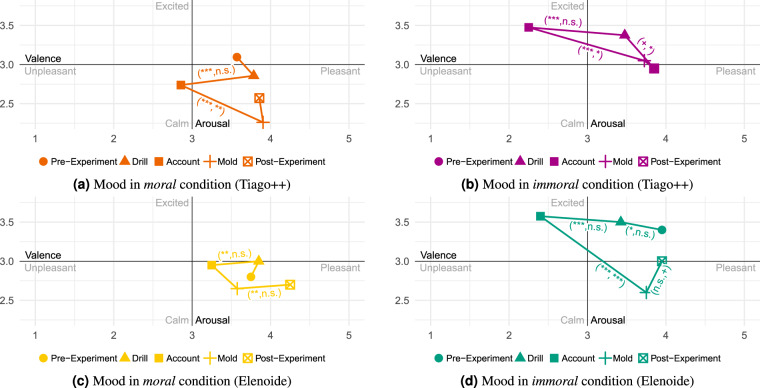
Mean mood differences over all participants in the *moral* and *immoral* conditions (Tiago++ vs. Elenoide) across all scenes. Only if a significant change in valence or arousal between two scenes is observed the tuple (significant valence change, significant arousal change) depicting the corresponding significance level is shown (*Note:* not significant (n.s.): p ≥ 0.1; ^+^p < 0.1; ^*^p < 0.05; ^**^p < 0.01; ^***^p < 0.001).

**Fig. 14:**
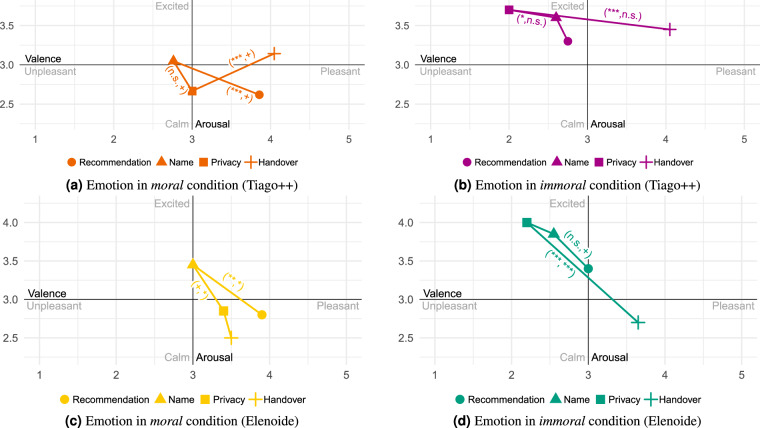
Mean emotional differences over all participants in the *moral* and *immoral* conditions (Tiago++ vs. Elenoide) across all scenes. Only if a significant change in valence or arousal between two scenes is observed the tuple (significant valence change, significant arousal change) depicting the corresponding significance level is shown (*Note:* not significant (n.s.): p ≥ 0.1; ^+^p < 0.1; ^*^p < 0.05; ^**^p < 0.01; ^***^p < 0.001).

To analyze the conditions’ effect on the participants’ subjective affect, the questions presented in Table 5 had to be answered using the SAM scales. Here, the questions about mood regarding a scene (e.g., *drill*) can be summarized into one construct. Looking at the relative changes between scenes or emotional triggers should make it possible to observe a causal effect if the condition was successful. By using the difference-in-difference technique, this effect can be determined by comparing the changes in mood (scenes) and emotions (triggers) of two conditions during the interaction period. In the analysis conducted here, the causal effects *δ* are considered relative to the previous scene/trigger, with the reported affective state from the pre-questionnaire as the starting point. The participants’ averaged mood during the scene and emotions at the triggers, respectively, are shown in Figs. 11, 12, 13, and 14. The annotations next to the lines represent the significant relative changes in valence or arousal between scenes and emotional triggers. The results of the difference-in-difference analysis are shown in the tables beside the sub-figures and in Table 8.

**Table 8:** Differences-in-differences effects of *moral* and *immoral* conditions (Tiago++/Elenoide) on human affect.

Scene Change	Moral-Immoral		Moral-Immoral		Neutral-Moral		Neutral-Immoral	
	(Tiago++)		(Elenoide)		(Tiago++)		(Tiago++)	
	*δ*_*valence*_	*δ*_*arousal*_	*δ*_*valence*_	*δ*_*arousal*_	*δ*_*valence*_	*δ*_*arousal*_	*δ*_*valence*_	*δ*_*arousal*_
Pre → Drill	0.589^*^	−0.663^+^	0.625^+^	0.1	−0.419	0.715^*^	0.171	0.052
Drill → Acc.	0.296	−0.219	0.425^*^	−0.125	−0.049	0.119	0.248	−0.1
Acc. → Mold	−0.427^+^	−0.051	−1.025^***^	0.675^*^	0.339	0.044	−0.089	−0.007
Mold → Post	−0.173	0.410	0.475^+^	−0.35	−0.021	−0.264	−0.193	0.146
Trigger Change								
Rec. → Name	−0.945^**^	0.129	−0.45	0.2	0.050	0.071	−0.896^**^	0.2
Name → Priv.	0.838^*^	−0.481^+^	0.75^*^	−0.75^*^	−0.875^*^	0.245	−0.036	−0.236
Priv. → Hand.	−1.002^*^	0.726^+^	−1.35^**^	0.95^**^	1.134^***^	−0.840^*^	0.132	−0.114

(*Note:* not significant (n.s.): p ≥ 0.1; ^+^p < 0.1; ^*^p < 0.05; ^**^p < 0.01; ^***^p < 0.001).

#### Transparency

Looking at the results regarding the *transparency* condition (compared with *neutral*) in Figs. 11 and 12, a significant causal effect in the valence scale is detected when changing from scenario *Account* to *Mold* or changing from the emotional trigger *Name* to *Privacy*, and *Privacy* to *Handover*. When considering the plots, this can be interpreted as during the *transparency* condition, the unpleasant feeling regarding disclosing personal data and coping with privacy information could be reduced. Overall, this condition resulted in a more relaxed and positive affect.

#### Liability

For the *liability* condition (compared with *neutral*), the significant causal effect shows that the failing interaction reduced the pleasant feelings (valence) during the mold interaction and, in particular, reduced the valence increase of performing the *Handover* by simultaneously having a tendency of increased arousal. This shows that, compared to the *neutral* condition, the failing handover interaction had a significant negative influence on the users’ affect.

#### Morality and Immorality

Comparing the *neutral* condition of Tiago++ with the *moral* condition regarding mood (see Figs. 11a and 13a), one can observe that the *moral* condition reduces arousal, also resulting in more relaxed arousal state. This is underlined by the significant emotion differences compared to *neutral* (see Fig. 12a vs. Fig. 14a), resulting in lower arousal and higher valence with the beginning of asking for *Privacy* information in the *moral* condition.

The *immoral* and *neutral* conditions do not show a significant difference in mood (Table 8). However, the *immoral* condition stays on a higher arousal level, which can be seen comparing Figs. 11a and 13b. A similar result can also be found for the emotional triggers.

Comparing the *moral* and *immoral* behavior during the interaction with Tiago++, a significant reduction in valence for *immoral* can be observed changing from *Pre-Experiment* to *Consultation* regarding mood (see Fig. 13). For the emotional triggers, the significant differences in valence show that the overall valence level drops not as much as in the *immoral* behavior. Further, the *immoral* behavior resulted in values with higher arousal levels for mood and emotions. Thus, being more friendly in the *moral* condition created a lower arousal than in the *immoral* condition.

The same relaxed affective mood state can also be observed, comparing the *moral* and *immoral* behavior with Elenoide. Here, the significant difference in valence and arousal when changing from *Account* to *Mold* shows the higher arousal and lower valence level before the mold scene at the *immoral* condition.

In summary, a more relaxed affect is perceived in the *moral* condition compared to the *immoral* and *neutral* one. Further, the *immoral* condition evokes more aroused and even stressed feelings than the *moral* and *neutral* conditions.

## Usage Notes

The published data set is the first to support the investigation of human affect in HRI, including labeled physiological data. We conducted a complex, realistic HRI study in a retail scenario, differentiating five conditions and three scenarios (see Fig. 9). Our data set includes physiological signals, robot behavior information (i.e., speech and gestures), and self-report questionnaire data regarding human affect, transparency, liability, and demography, collected from 146 participants. The data set can be used to study and improve emotion and mood recognition, robot behavior in a retail environment, and liability and transparency in an HRI as described in the following subsections.

### Emotion and mood recognition

The physiological sensor data as an affective response to the robot’s behavior can be used to prove established or develop new emotion recognition methods and technological capabilities for HRI. In the Technical Validation Section, we have already shown the causal effects of the different conditions on the participant’s affect. Therefore, utilizing our data set for research in affective computing is valuable. The *ground_truth.csv* file can be merged with the participants’ sensor data with the help of the NTP timestamps. The timestamps of the robot utterance and gesture do not coincide with those of the recorded physiological data, and therefore a matching using the timestamps is not possible. For that, we recommend using an ordered merge on the NTP timestamp column. After this ordered merge, the resulting data structure contains for each timestamp an entry that includes all columns of the *ground_truth.csv* file and physiological sensor file. Here, the additional columns of the counterpart are filled with *NaN* or *NULL* values. This means, that rows originating from the physiological data file are extended with columns from the *ground_truth.csv* file (such as gesture and speech) filled with *NaN* or *NULL*. The rows originating from the *ground_truth.csv* file are accordingly extended with a column of the physiological data filled with *NaN* or *NULL*. Except for the emotion columns, these *NaN* or *NULL* values can be replenished by performing a forward fill on all columns from the *ground_truth.csv* file. As emotions are short-term reactions (0.5–4 seconds^7^; see Section Questionnaire Emotion and Mood), the labels for the emotion should be applied to the physiological signal in accordance with this reaction time. The forward fill and labeling assigns labels to the sensor data without changing the physiological data column. This maintains the sensors original sampling rate. Finally, the original rows of the robot’s utterance and gesture can be deleted to avoid *NaN* or *NULL* values in the physiological signals columns.

After that, the data can be classified and analyzed utilizing Python packages, like Pandas, Scipy, or NumPy libraries. As we publish raw data, we recommend pre-processing the data before usage. Python libraries such as Neurokit2^50^, HRVanalysis^52^, or cvxEDA^53^ are particularly suitable for data cleaning, feature extraction, change-point detection, and data analysis. Further information on processing and using the sensor data can be found on the manufacturer’s website (Recommended tools for signal processing and data analysis). Through the use of the SAM scale^12^ and physiological data gathered by the E4 wristband, a combination with other open data sets, containing the same modalities (e.g., WESAD^16^, Angry or Climbing Stairs^15^) is possible. Thus, more sophisticated research on physiological sensor data labeled by human affect can be achieved.

### Robot behavior in a retail environment

**Fig. 15:**
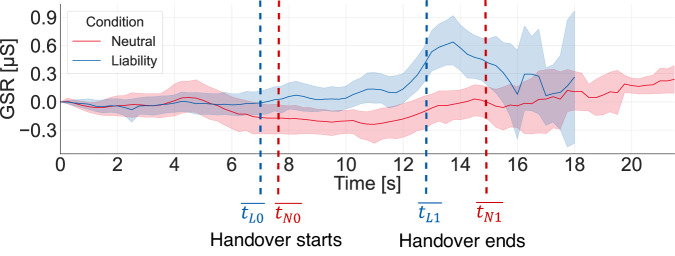
Mean GSR signal during the handover.

We call for an in-depth investigation regarding specific robot behavior (i.e., gestures and speech) on the human affect, considering the whole range of provided behavioral nuances across all conditions and scenes. Preliminary analyses^37^ showed, for example, that different conditions had an effect on the physiology of the participants, such as the failing handover signal in the *liability* condition (see Fig. 15). But this handover may also be perceived differently by the participants depending on the robots interaction behavior across the other conditions. Furthermore, the various available speech texts of the robots (listed in *gesture_speech.csv*) can also be analyzed for emotion transmission using Natural Language Processing (such as Linguistic Inquiry and Word Count (LIWC)^54,55^). The effects of emotions transmitted via the robot’s utterance could have influenced the participants’ affect and their answers to the questionnaire (per participant stored in *ground_truth.csv*).

### Liability and transparency in an HRI

The presented data set provides the possibility to evaluate the HRI regarding our participants’ perceived liability and transparency (available in *questionnaire.csv*). In a previous publication^37^, a first impact on this was shown. However, analyzing and evaluating the data among the conditions, such as liability issues, is worthwhile. For example, the assessed liability expectations could have been changed by the *moral* or *immoral* robot behavior. This effect is also of importance, as liability might influence the robot’s behavioral design in the future^37^. For this purpose, regression models can be used, which can be controlled, e.g., for age. For further statistical investigations, the *questionnaire.csv* file can be analyzed using statistical software such as R, SPSS, or Stata.

### Discussion and limitations

The previous evaluations of the published data set show that a cross-disciplinary view of the data set is a win for robot design^10,37^. Our interdisciplinary research team developed five different conditions (i.e., *neutral*, *transparency*, *liability*, *moral*, and *immoral*) and proved the conditions’ causal effect on the perceived affective state (see Section Statistical Validation of Conditions). Thus, the use of the conditions is valid to be considered for further research. By evaluating the data set with the GARAFED method, we showed that the data set meets high requirements. Even though our data set includes 146 participants, an imbalance in sociodemographic characteristics, technical affinity, and experience with robots can be observed, caused by our advertisement (email to the Technical University of Darmstadt’s employees and students). Especially the differences in the participant’s age per condition should be addressed by adding a control variable in the statistical evaluation to generalize results. A diverse range of demographics in emotion recognition helps to generalize emotion recognition models to a larger population, enhancing the reliability and accuracy of the model^56^. Accordingly, results generated from this data set must be set in context. Further, many people struggle to correctly rate or name their affective state^9^, causing a bias. This bias can be amplified by asking the participants to rank their affective state after the HRI, as they might not correctly remember it. On the other hand, asking after the HRI has the benefit of not disturbing the interaction. Thus, we suggest the additional use of physiological sensor data to underline the participant’s affective state as an objective measure. Furthermore, using the baseline measurements might also include biases: the first baseline measurement might be biased by the participant’s pre-measurement expectations, whereas the second measurement might be biased by what was experienced. Thus, to mitigate these single biases, both baseline measurements are provided to create a more reliable baseline. Further, the change in the participant’s pre- and post-state within the physiological data is implicitly provided by the first and second baseline measurements. As outlined in Section Emotion and Mood Recognition, the physiological signals and gathered ground-truth data can be combined. However, this includes some uncertainties. One of these uncertainties is the unknown duration of the robot’s utterances and gestures. Accordingly, the values from the physiological signals cannot be unambiguously linked to the robot’s utterance and gestures, and the time at which the test person spoke is not recognizable. Nonetheless, we decided to link the physiological signals with the previously performed utterance or gesture, as it may influence the subsequent physiological signals. Another uncertainty is whether the presupposed emotional triggers served as such for each individual. To prove whether these emotional triggers elicited emotions, breakpoint detection or peak detection in the physiological signal could help to find changes, indicating an effect on the physiological signals. These changes could be placed in the context of possible emotional triggers. Nevertheless, we want to encourage other researchers to use our data set to investigate this challenge of labeling physiological signals correctly with data gathered within a questionnaire. Another limitation arises from the questionnaire addressing liability, which was explicitly created for this study. The assessed parties (e.g., robot, customer, hardware store) are generalized and were chosen to simplify the questionnaire. Regardless, this simplification does not distinguish between specific parties. For instance, the term robot generally refers to the provider of certain parts (e.g., software), manufacturer, seller, or robot. Therefore, a distinction between these particular parties is not possible with our data. Accordingly, when evaluating this part of the questionnaire, the results obtained will be of a more general nature.

Future work could extend the scope of the research, by performing a human-human interaction in a similar scenario. The comparison with a human-human interaction seems promising to evaluate whether the interaction with a human or robot has different effects on the human affective state^10^. Furthermore, conducting a crossover study^57^ concerning the change in the suggested task sequence could provide further insights into the single effects of the tasks on changes in the affective state.

In summary, the call of previous scientific papers^3–6^ for more published data sets is answered by us, publishing physiological data labeled by human affect as ground truth. We wish this data set will be evaluated comprehensively and contribute significantly to further developments of emotion recognition in HRI.

### Limitation on data use

This work is licensed under CC-BY 4.0 You will uphold participants’ privacy in this data set by not attempting to re-identify the participants.

### Additional information

The depicted individual in the figures, and the photographer permitted to publish the images by signing a GDPR compliant consent form.

## Data Availability

The published data set contains raw anonymized data. All anonymization steps can be found in Section Data Anonymization. The SAM mapping can be found in Questionnaire Emotion and Mood. No additional code was used to generate the data set.
